# The Efficacy and Safety of Diyushengbai Tablet on Preventing and Treating Leukopenia Caused by Radiotherapy and Chemotherapy Against Tumor: A Systematic Review and Meta-Analysis

**DOI:** 10.3389/fphar.2022.827710

**Published:** 2022-07-19

**Authors:** Xiaoqing Xu, Hongmin Li, Xiwen Hu, Juan Liu, Fang Zhao, Kaiyong Xie, Jun Wang

**Affiliations:** ^1^ Department of Oncology, Affiliated Hospital of Shandong University of Traditional Chinese Medicine (Shandong Provincial Hospital of Traditional Chinese Medicine), Jinan, China; ^2^ Oncology Center, Sichuan Academy of Medical Sciences and Sichuan Provincial People's Hospital, Chengdu, China; ^3^ Chengdu Di’ao Group Tianfu Pharmaceutical Co., Ltd., Chengdu, China; ^4^ Department of TCM Pharmacy, Chengdu Integrated TCM and Western Medicine Hospital, Chengdu, China

**Keywords:** Diyushengbai tablet, tumor, radiotherapy and chemotherapy, meta-analysis, systematic review

## Abstract

**Background:** Leukopenia is one of the side effects of radiotherapy and chemotherapy. Diyushengbai tablet (DYT) is used to prevent and treat leukopenia caused by various reasons. A meta-analysis was performed to systematically analyze the therapeutic effects of DYT on preventing and treating leukopenia caused by radiotherapy and chemotherapy.

**Objectives:** This study aimed to systematically evaluate the efficacy and safety of DYT in preventing and treating leukopenia caused by radiotherapy and chemotherapy.

**Methods:** We performed a comprehensive literature search of electronic databases such as PubMed, The Cochrane Library, China Knowledge Network (CNKI), China Biomedical Literature Database (CBM), Wanfang Data Knowledge Service Platform, and VIP, through November of 2021. The scanning reports deadline is until November 2021. The bias risk evaluation criteria developed by the Cochrane collaborative organization were used to evaluate the literature quality of the included studies. The RevMan5.4 software was used to analyze the data, and the Stata16.0 was used to perform the Egger test.

**Results:** After selecting all the databases, a total of 41 reports which involved 3,793 cases were analyzed. Meta-analysis showed that DYT could significantly reduce the white blood cell (WBC) suppression caused by radiotherapy and chemotherapy and improve the patients’ WBC counts and neutrophils, compared with the efficacy of other oral WBC-elevating drugs such as Leucogen tablets and Batilol tablets and additional utilization of granulocyte colony-stimulating factor (G-CSF). The results of meta-analysis showed that for preventive medication purpose, the overall incidence of leukocyte suppression was [RR = 0.74, 95%CI (0.59, 0.92), *p* = 0.006], and the white blood cell count was [MD = 1.12, 95%CI (0.95, 1.29), *p* < 0.00001]; while for therapeutic purpose, the incidence of overall leukocyte suppression was [RR = 0.61, 95%CI (0.38, 0.95), *p* = 0.03], and the white blood cell count was [MD = 1.20, 95%CI (0.77, 1.62), *p* < 0.00001]. More importantly, the additional use of DYT can reduce the application amount of G-CSF. The results showed that the application of G-CSF can be reduced by an average of 1.57 from the beginning of treatment to return normal white blood cells around 2.23 in two cycles of chemotherapy.

**Conclusion:** DYT is more effective in preventing and treating leukopenia caused by radiotherapy and chemotherapy than other oral WBC-elevating drugs, which have a high clinical value.

## 1 Introduction

Malignant tumor is a common disease that can be harmful to people’s health. Radiotherapy and chemotherapy are the effective as well as primary treatment methodologies. At the same time, there are many side effects during radiotherapy and chemotherapy due to the use of radiation and chemotherapeutic drugs (Chen et al., 2014; Tian et al., 2018). Leukopenia is one of the most common side effects, with a high incidence of 30%–50% (Li et al., 2015). It can affect the normal progress of treatment and even lead to failure because patients can be infected seriously.

Diyushengbai tablet (DYT) produced by Chengdu Di’ao Group Tianfu Pharmaceutical Co., Ltd. is used to prevent and treat leukopenia caused by various reasons. Its main active ingredient is Sanguisorbae Radix recorded in Chinese Pharmacopoeia as the dry roots of *Sanguisorba officinalis* L. or *Sanguisorba officinalis* L. var. *longifolia* (Bert.) Yü et Li (Pharmacopoeia Commission Chinese, 2020). Many studies described that DYT could be used to prevent and treat myelosuppression caused by radiotherapy and chemotherapy in recent years. Also, it could raise the WBC counts as well as reduce the application amount of G-CSF effectively with no significant toxicity and side effects (Shi et al., 2018; Zhang and Shuai, 2019). A systematic review and meta-analysis were conducted to further figure out the efficacy and safety of DYT for preventing and treating leukopenia and provide more credible proof of evidence-based medicine.

## 2 Materials and Methods

### 2.1 Search Strategy

A search of PubMed, EMBASE, The Cochrane Library, China Knowledge Network (CNKI), China Biomedical Literature Database (CBM), WanFang Data Knowledge Service Platform, and VIP Database for Chinese Technical Periodicals (VIP) for trials up to November 2021 was conducted. We used full-text search keywords;. the Chinese search terms included “Diyushengbai tablet (地榆升白片)” while the English term was “Diyushengbai tablet” or “Diyushengbaipian.”

### 2.2 Inclusion and Exclusion Criteria

Inclusion criteria: 1) Chinese or English studies of randomized controlled trials (RCTs) with subjects receiving radiotherapy and/or chemotherapy. The treatment group took DYT or combined with G-CSF, while the control group received placebo, other WBC-elevating drugs, G-CSF alone, or no treatment. 2) Studies were reported with specific and intact data including basic information of each group, number of cases, interventions, treatment courses, clinical outcomes, etc. 3) Clinical outcomes included white blood cells (or neutrophils) or suppression rate. Both WBC and blood platelet suppression rate refer to WHO adverse events degree criteria, while the WBC effective rate refers to the criteria in Guideline for Traditional Chinese Medicine Clinical Practice enacted by Ministry of Health of the People’s Republic of China, which are as follow: marked effectiveness, for the total amount of WBC >4.0 × 10^9^/L and last for a week after the withdrawal with remarkable remission or disappearance of clinical symptoms; general effectiveness, for the total amount of WBC <4.0 × 10^9^/L, but increase (0.5–1.0) × 10^9^/L compared to before, and last for a week after the withdrawal with the improvement of clinical symptoms. Ineffectiveness, for the increment of WBC <0.5 × 10^9^/L. It is also feasible to refer to other criteria in accordance with the above.

Exclusion criteria: 1) Cases complicated with any serious internal diseases, such as cardiac, cerebral, and renal injury; 2) the design of the research was combined with other WBC-elevating treatments; 3) there was no control group or self-control only; 4) articles were about cohort study, animal experiment, clinical experience, etc.; 5) conference articles; 6) graduation papers.

The intervention was split into three subgroups according to different treatments of the control group: Subgroup 1, no application of any other WBC-elevating drug in the control group; Subgroup 2, application of at least one WBC-elevating drug except DYT; Subgroup 3, application of G-CSF or G-CSF as needed. The purpose of medication was divided into preventive and therapeutic, according to the intervention time of DYT therapy. For preventive medication, the treatment intervened before or at the same time of the radiotherapy and chemotherapy with WBC within the normal range, while as for therapeutic medication, the treatment intervened after the radiotherapy and chemotherapy with WBC under the normal range. The diagnostic criterion of leukopenia was that the WBC count was less than 4.0 × 10^9^/L continuously for at least 2 weeks.

### 2.3 Risk Assessment and Data Extraction

Studies were assessed, and data were extracted by two researchers, respectively. Once disagreements were aroused, the third researcher would get into the discussion and make the final decision. According to the well-designed data extraction form, information of studies was collected, which included authors, year of publication, number of cases, interventions, clinical outcomes, allocation methods, etc.

The Cochrane Risk of Bias Tool was used to evaluate the literature quality of the included studies, including randomization, allocation concealment, blinding, withdrawal, and loss to follow-up. Two researchers cross-checked the evaluation results, and once their opinions did not meet, the third researcher would help make the final call. Original authors were contacted to obtain the missing data in necessity.

### 2.4 Analysis Methods

RevMan5.4 was used to analyze the data and draw the funnel plots. Risk ratio (RR) was the effect size of binary variables, while mean difference (MD) was the effect size of continuous variables. 95% confidence interval was calculated as well. Heterogeneity among studies was assessed using the Q test and the I^2^ value to determine the degree of heterogeneity. The fixed effected model was adopted when studies showed a high homogeneity (*p* > 0.05, I^2^ < 50%); otherwise, meta-regression was used to analyze the sources of between-study heterogeneity, and a random-effects model was adopted. Sensitivity analysis was conducted to verify the stability of results by eliminating each study individually. Egger's test was used to evaluate whether each study had a publication bias. Egger’s test, sensitivity analysis, and meta-regression analysis were completed with Stata SE 16.0.

## 3 Results

### 3.1 Study Selection and Study Characteristics

**FIGURE 1:**
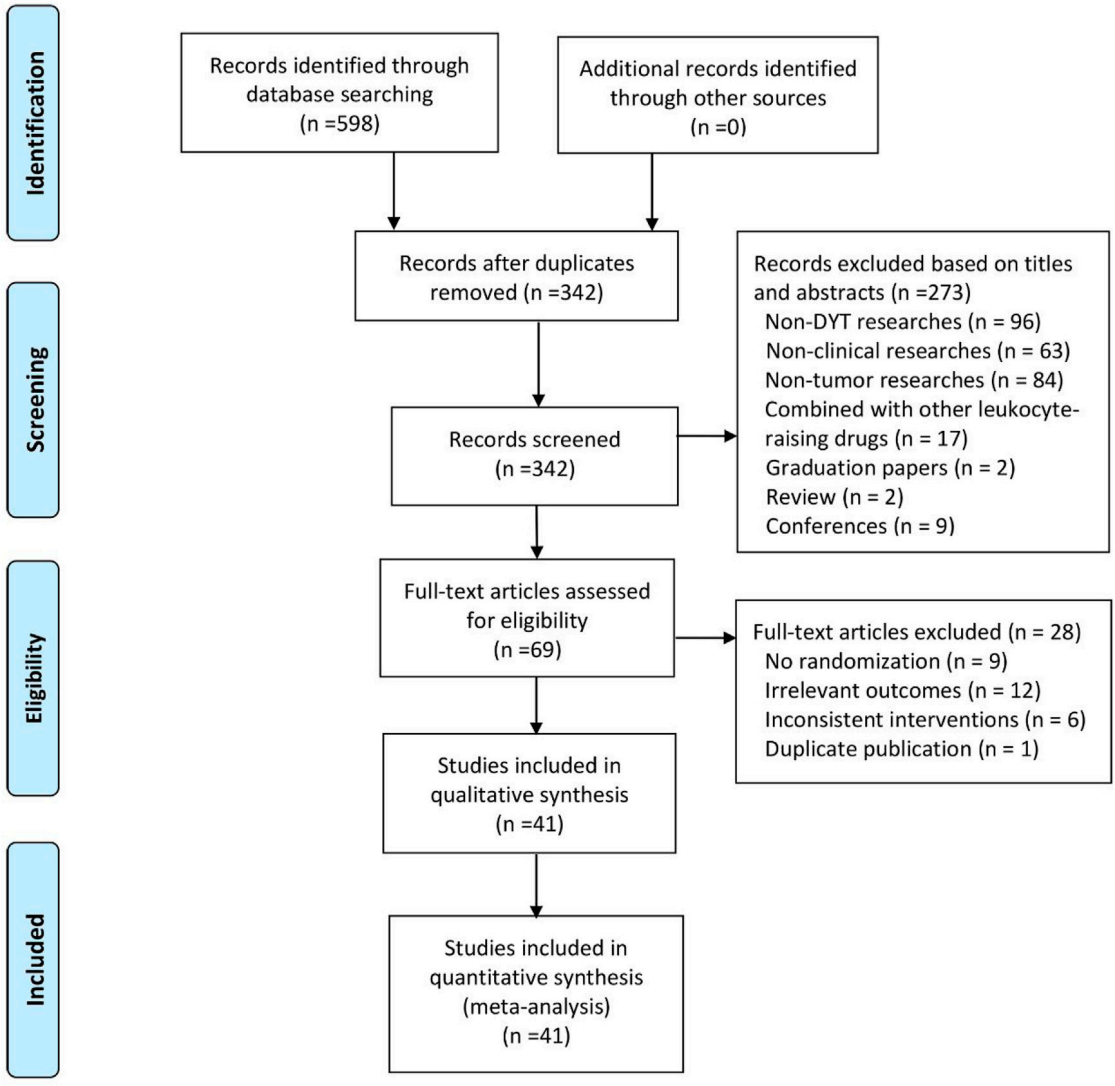
Technology roadmap of bibliographic retrieval.

A total of 598 articles were retrieved. Overall, 342 articles remained after deleting duplicate literature. Based on the titles and abstracts, an additional 273 studies were excluded for they were either non-clinical research, irrelevant research direction, combined with other drugs of raising leukocyte, animal experiments, graduation papers, or review. There were 69 full-text articles left, from which we excluded 28 trials because they were not randomized, had irrelevant outcomes, had inconsistent interventions, or were duplicate publications. Therefore, a final total of 41 studies (Wang et al., 2003; Xu et al., 2003; Li et al., 2004; Wang et al., 2003; Ma, 2005; Xu et al., 2005; Zhu, 2005; Chen et al., 2006; Chen2 et al., 2006; Wang et al., 2006; Wang2 et al., 2006; Xu et al., 2006; Xu, 2006; Yin amd Kang, 2006; Li and Yang., 2007; Zeng et al., 2007; Zhao et al., 2007; Dong and Huang, 2010; Gong and Duan, 2010; Hu et al., 2010; Wang et al., 2010; Hui et al., 2011; Li et al., 2011; Liu, 2011; Wu, 2011; Yin et al., 2011; Li and Yang, 2012; Feng et al., 2013; Wang, 2013; Fu, 2014; Zhang, 2014; Jiang et al., 2015; Ming and Zhang, 2015; Weng et al., 2015; Chen, 2016; Zhao et al., 2017; Wang et al., 2017; Deng et al., 2018; Shi et al., 2018; Mou et al., 2019; Weng and Zeng, 2019) were included in our research (4-43), 19 of which were for therapeutic and the rest of which were for preventive (Figure 1)—involving 3,793 cases, among which 1,954 were allocated to the treatment group while the other 1,839 were in the control group. The names of the studies, first authors, years of publication, numbers of enrolled patients, experimental intervention and control groups, treatment time, and outcome data were extracted (Table 1).

**TABLE 1:** Information of included studies.

Included studies	No.(T/C)	Treatment group	Control group	Course of treatment	Dose of DYT	Antitumor interventions	Clinical outcomes	Medication purpose	Subgroup
Xiangbo Wang et al. (2003)	35/34	DYT	—	4 weeks	0.3 g tid	radical RT	①③④	Preventive	1
Ninghong Xu et al. (2003)	66/62	DYT	Leucogen tablets	Till WBC level back to normal	0.2–0.4 g tid	RT and/or CT	⑥	Therapeutic	2
Youming Li et al. (2004)	33/30	DYT	G-CSF as needed	Till WBC level ≥5 × 10^9^/L	0.4 g tid	CT	①③④⑤	Preventive	3
Shijin Wang (2004)	72/48	DYT	—	3 weeks	0.3 g tid	CT	①⑥	Therapeutic	1
Qiang Ma (2005)	90/90	DYT	Batilol tablets	Same as the chemoradiotherapy	0.3 g tid	RT and/or CT	①③④	Preventive	2
Xinhua Xu et al. (2005)	58/49	DYT	Vitamin B4 + Leucogen tablets	6 weeks	0.2 g tid	radical RT	①②④⑤	Preventive	2
Yu Zhu (2005)	62/58	DYT	Vitamin B4	20 days	0.3 g tid	radical RT	①	Therapeutic	2
Yong Chen et al. (2006)	50/46	DYT	Leucogen tablets	60 days	0.4 g tid	CT	⑥	Therapeutic	2
Zhiming Chen (2006)	28/32	DYT	G-CSF as needed	Till WBC level ≥5 × 10^9^/L	0.4 g tid	CT	①⑤⑦	Preventive	3
Wei Wang (2006)	40/38	DYT	Leucogen Tablets	4 months	0.3 g tid	CT	⑤	Preventive	2
Zhongsu Wang (2006)	65/40	DYT	Leucogen tablets	6 weeks	0.4 g tid	RT and/or CT	⑤⑥	Therapeutic	2
Xiaodong Xu (2006)	72/86	DYT	Leucogen tablets + Batilol tablets	Same as the chemoradiotherapy	0.4 g tid	RT	⑤	Preventive	2
Hong Xu (2006)	45/40	DYT	Batilol tablets	60 days	0.3 g tid	CT	⑥	Therapeutic	2
Liang Yin and Kang (2006)	55/55	DYT	Leucogen tablets + Batilol tablets	3 weeks	0.2–0.4 g tid	CT	⑥	Therapeutic	2
Hong Li and Yang, (2007)	46/42	DYT	Leucogen tablets + Batilol tablets	40 days	0.4 g tid	CT	⑦	Preventive	2
Zhaoyu Zeng (2007)	32/37	DYT	G-CSF as needed	Till WBC level ≥5 × 10^9^/L	0.4 g tid	CT	①⑤⑦	Preventive	3
Weiyong Zhao (2007)	30/30	DYT + G-CSF	Leucogen tablets + G-CSF	Same as the chemoradiotherapy	0.4 g tid	RT	⑤	Therapeutic	3
Ying Dong and Huang, (2010)	33/30	DYT	G-CSF as needed	Till WBC level ≥10 × 10^9^/L	0.4 g tid	CT	①④⑦	Preventive	3
Jianyi Gong (2010)	47/45	DYT	G-CSF as needed	3 weeks	0.4 g tid	CT	⑤	Preventive	3
Qian Hu (2010)	17/16	DYT	G-CSF as needed	Till WBC level back to normal	0.4 g tid	CT	①③④	Preventive	3
Fei Wang (2010)	120/100	DYT	Leucogen tablets	2 months	0.3 g tid	CT	⑥	Therapeutic	2
Hui Shuang (2011)	40/40	DYT	G-CSF as needed	6 weeks	0.4 g tid	CT	⑤⑦	Preventive	3
Zhigang Li (2011)	36/34	DYT	G-CSF as needed	Till WBC level back to normal	0.4 g tid	CT	⑤⑦	Therapeutic	3
Yangfan Liu (2011)	65/68	DYT	Leucogen tablets	Same as the chemoradiotherapy	0.4 g tid	RT	⑤	Preventive	2
Yangdong Wu (2011)	35/33	DYT	G-CSF as needed	Till WBC level ≥5 × 10^9^/L	0.4 g tid	CT	①⑤	Preventive	3
Xiaodong Yin (2011)	45/45	DYT	Leucogen Tablets	Till WBC level back to normal	0.4 g tid	CT	⑤	Preventive	2
Qiumei Li (2012)	45/42	DYT	—	Same as the chemoradiotherapy	0.3 g tid	RT and/or CT	①⑤⑦	Preventive	1
Chun Feng (2013)	26/22	DYT	G-CSF as needed	16 weeks	NR	CT	①⑤	Therapeutic	3
Renxiao Wang (2013)	20/20	DYT + G-CSF	G-CSF as needed	63 days	NR	CT	①②	Therapeutic	3
Liran Fu (2014)	35/32	DYT	Batilol tablets + Vitamin B4	21 days	0.3 g tid	RT	①⑥	Therapeutic	2
Jingjing Zhang (2014)	60/60	DYT	Leucogen tablets	60 days	0.3 g tid	CT	⑥	Therapeutic	2
Danxian Jiang (2015)	30/29	DYT	—	21 weeks	0.4 g tid	CT	①③④	Preventive	1
Bangchun Ming (2015)	45/45	DYT	G-CSF as needed	2–3 weeks	0.3 g tid	RT and/or CT	①⑤⑥	Therapeutic	3
Yijie Weng (2015)	32/29	DYT	—	18 weeks	0.4 g tid	CT	②⑧	Preventive	1
Yuanqian Chen (2016)	37/41	DYT + G-CSF	G-CSF as needed	21 days	0.4 g tid	RT and/or CT	①②	Therapeutic	3
Hong Qiao (2017)	50/50	DYT	Batilol tablets	60 days	0.3 g tid	CT	⑥	Therapeutic	2
Wenjuan Wang (2017)	100/84	DYT + G-CSF	G-CSF as needed	Till WBC level back to normal	0.2 g tid	CT	①	Therapeutic	3
Bo Deng (2018)	40/40	DYT	—	5 weeks	0.4 g tid	adjuvant RT	②⑧	Preventive	1
Shi et al. (2018)	56/56	DYT	—	4 weeks	0.4 g tid	RT	①⑨	Preventive	1
Daying Mou (2019)	40/40	DYT	—	9 weeks	0.4 g tid	CT	①③④⑤⑨	Preventive	1
Jianfeng Weng (2019)	21/21	DYT + G-CSF as needed	G-CSF as needed	21 days	0.2–0.3 g tid	CT	①	Therapeutic	3

Note: No., number of participants; T, treatment group; C, control group; DYT, Diyushengbai tablet; RT, radiotherapy; CT, chemotherapy; NR, not reported. Outcome Indicators: ① white blood cell count; ② neutrophil count; ③ blood platelet count;④ hemoglobin count; ⑤ white blood cell suppression rate; ⑥ white blood cell effective rate; ⑦ application number of G-CSF; ⑧ immune factor; ⑨ tumor effective rate.

### 3.2 Literature Quality

**FIGURE 2:**
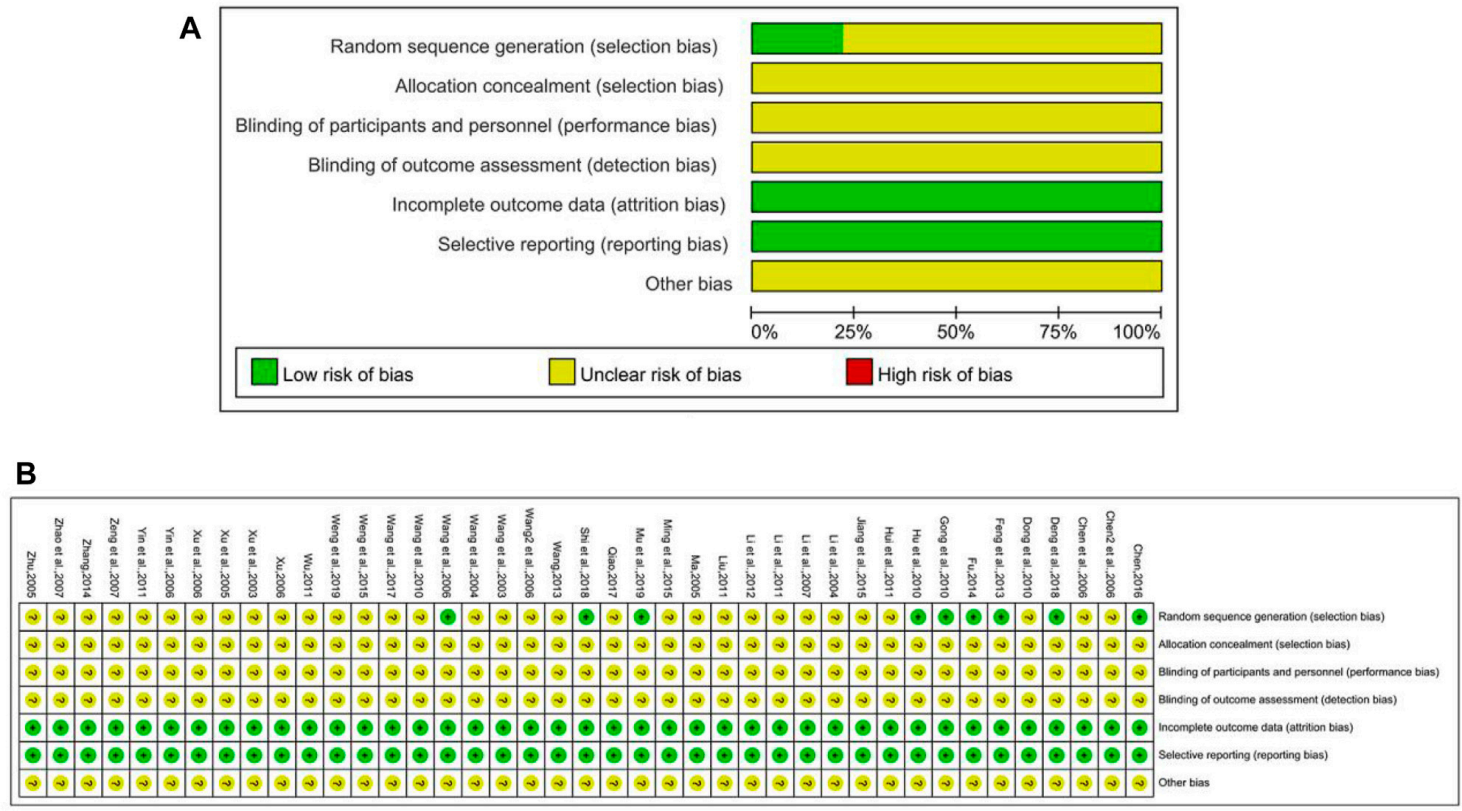
Risk of bias graph of Included Studies **(A)** Risk of bias graph **(B)** Risk of bias summary.

The risk of bias graph of included studies is shown in Figure 2. Among all 41 included RCT studies, 8 of them described the method for random sequence generation, in which 6 trials (Wang et al., 2006; Feng et al., 2013; Fu, 2014; Chen, 2016; Deng et al., 2018; Mou et al., 2019) used the random number table, 2 trails (Gong and Duan., 2010; Hu et al., 2010) adopted the sealed envelopes method, and 1 trail (Shi et al., 2018) used lottery, while the rest did not mention the randomization method. None of the researchers reported the method to conduct allocation concealment and blinding. In addition, withdrawal and loss to follow-up did not happen in any study.

### 3.3 Meta-Analysis Results of Preventive Medication

#### 3.3.1 White Blood Cell Count

**FIGURE 3:**
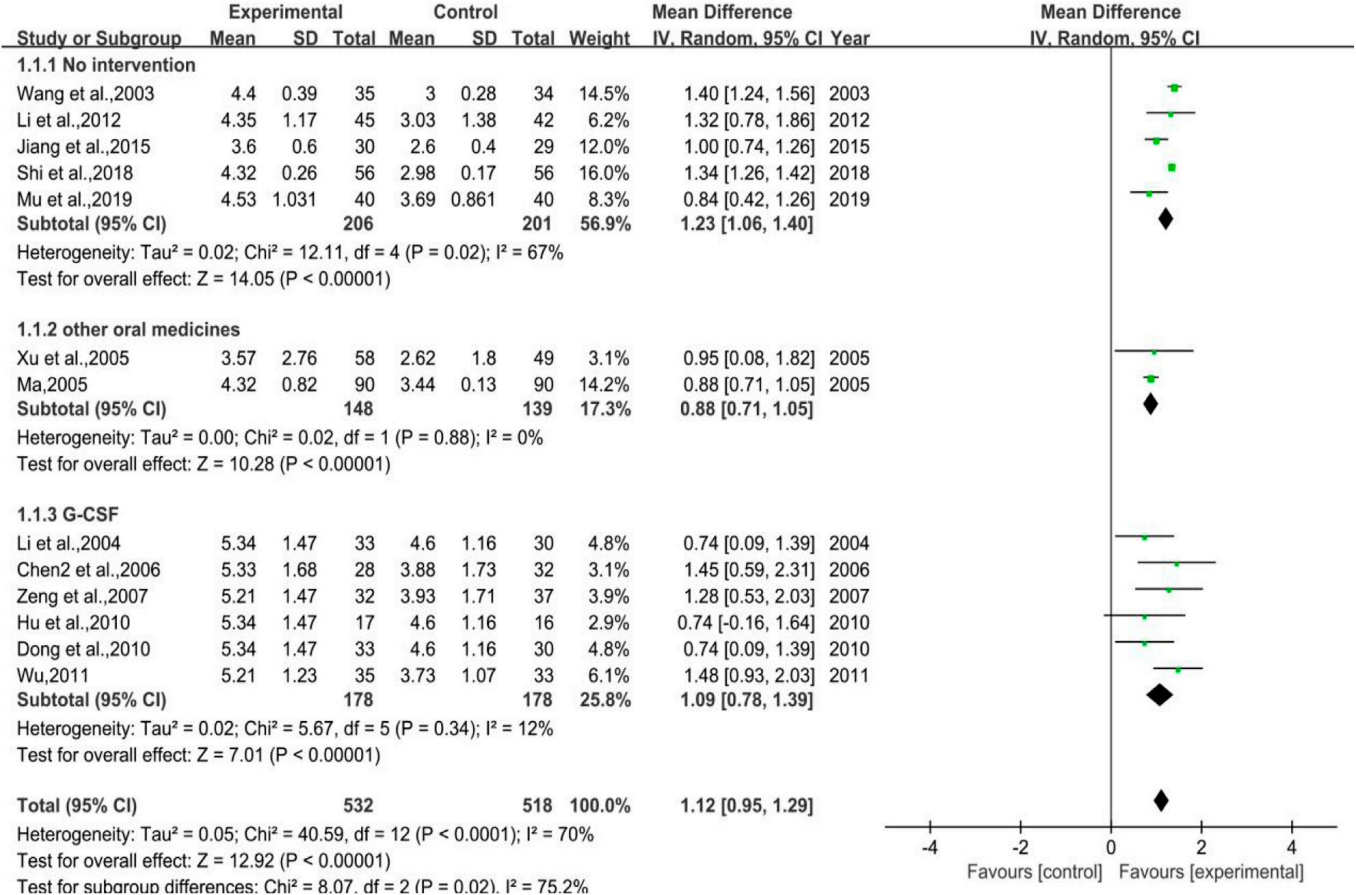
The forest plot of WBC count in preventive medication.

A total of 13 trials were included, of which five were with control group not receiving any treatment (Subgroup 1), two were exposed to other WBC-elevating drugs (Subgroup 2), and the rest were with G-CSF (Subgroup 3). The number of cases in treatment group and control group were 532 and 518, respectively. The heterogeneity differed in different groups for *p* < 0.0001, I^2^ = 70% in total; *p* = 0.02, I^2^ = 67% in Group 1. Meta-regression showed that the heterogeneity of group 1 was not significantly related to the years of publication (*p* = 0.451), the duration of medication (*p* = 0.831), the number of case (*p* = 0.102), anti-tumor treatment (*p* = 0.359), the dose of DYT (*p* = 0.109), study region (*p* = 0.491), hospital grade (*p* = 0.383); *p* = 0.88, I^2^ = 0% in Group 2; *p* = 0.34, I^2^ = 12% in Group 3. The results of meta-analysis showed that [MD = 1.12, 95% CI (0.95, 1.29)], Z = 12.92 (*p* < 0.00001) in total; [MD = 1.23, 95%CI (1.06, 1.40)], Z = 14.05 (*p* < 0.00001) in Group 1. Sensitivity analysis showed that the results were stable, and the removal of each study had little effect on the overall results (Figure 17A), and Egger’s test (*p* = 0.247) showed that the included studies were without significant publication bias; [MD = 0.88, 95%CI (0.71, 1.05)], Z = 10.28 (*p* < 0.00001) in Group 2; [MD = 1.09, 95%CI (0.78, 1.39)], Z = 7.01 (*p* < 0.00001) in Group 3. Sensitivity analysis showed that the results were stable, and the removal of each study had little effect on the overall results (Figure 17B), and Egger’s test (*p* = 0.687) showed that the included studies had no significant publication bias. The results implied that for preventive medication, the efficacy of DYT in improving WBC count was superior to the control group, the difference between two groups was statistically significant. The forest plot of meta-analysis is depicted in Figure 3.

#### 3.3.2 Neutrophile Count

**FIGURE 4:**
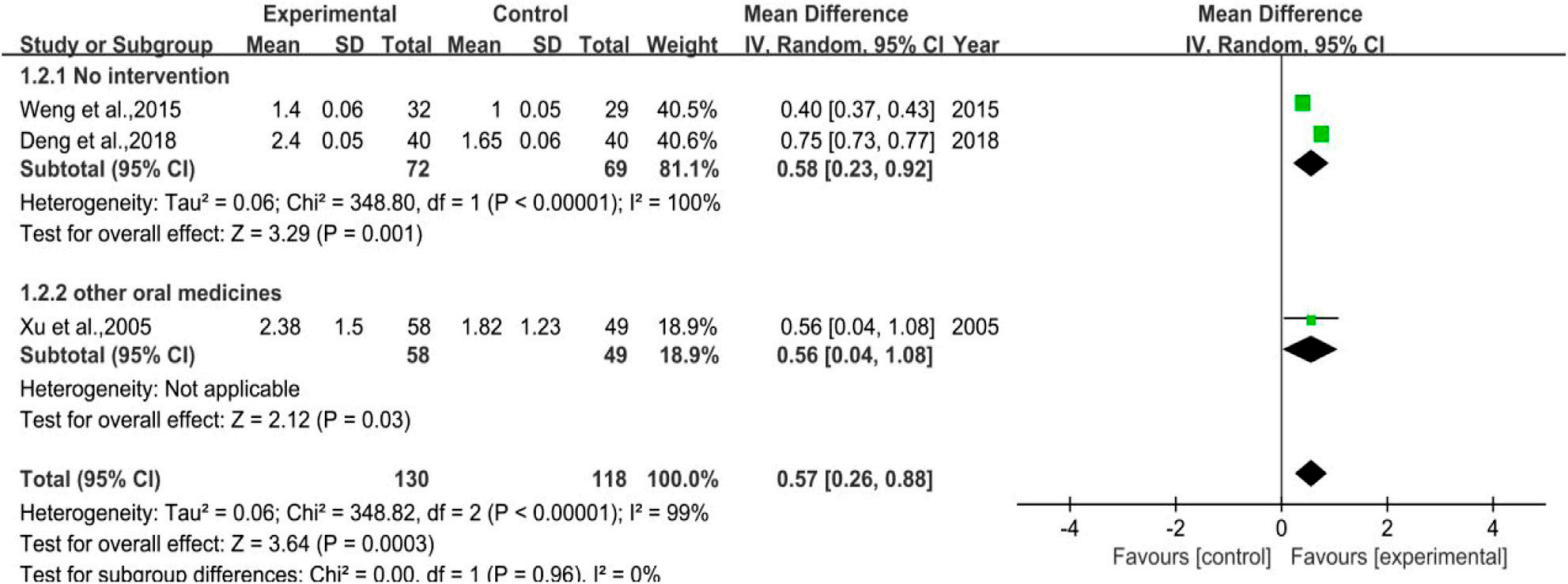
The forest plot of neutrophil count in preventive medication.

There were 3 trials involved in the analysis, two of them were studies in which no intervention was applied to the control group (Subgroup 1), and the other one was the control group used with other drugs (Subgroup 2). The number of cases included was 130 and 118 in the treatment and control groups, respectively. The heterogeneity results revealed that no matter in total or in Subgroup 1, the heterogeneity was quite high (*p* < 0.00001, I^2^ = 99% in total; *p* < 0.00001, I^2^ = 100% in Subgroup 1). Besides, for the overall effect in total, the meta-analysis result was [MD = 0.57, 95%CI (0.26, 0.88)], Z = 3.64 (*p* = 0.00003), while the result was [MD = 0.58, 95%CI (0.23, 0.92)], Z = 3.29 (*p* = 0.001) in Subgroup 1. All the results above hinted that for preventive medication, DYT performed better than the control group in raising neutrophile count, with statistically significant difference between the two groups. Figure 4 shows the forest plot of the meta-analysis.

#### 3.3.3 Platelet Count

**FIGURE 5:**
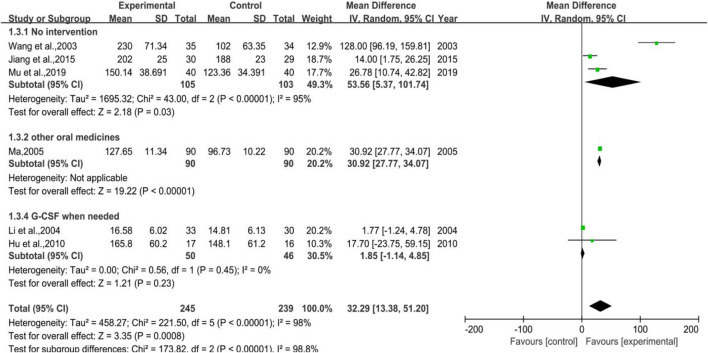
The forest plot of platelet count in preventive medication.

Figure 5 depicts vividly that 6 trials were enrolled in total, of which three belonged to Subgroup 1 (no intervention applied in control group), one pertained to Subgroup 2 (using other WBC-elevating drugs), and the other two were in Subgroup 3 (using G-CSF as needed). The number of cases in the treatment group were 245, while there were 239 cases in the control group. It was hinted that the total heterogeneity was *p* < 0.00001, I^2^ = 98%, and the heterogeneity of Subgroup 1 was *p* < 0.00001, I^2^ = 95%, while that of Subgroup 3 was *p* = 0.45, I^2^ = 0%. The results of meta-analysis were as listed, [MD = 32.29, 95%CI (13.38, 51.20)], Z = 3.35 (*p* = 0.0008) in total, [MD = 53.56, 95%CI (5.37, 101.74)], Z = 2.18 (*p* = 0.03) in Subgroup 1, [MD = 1.85, 95%CI (-1.14, 4.85)], Z = 1.21 (*p* = 0.23) in Subgroup 3. Overall, the efficacy of DYT was significantly superior to the control group in improving platelet count.

#### 3.3.4 Hemoglobin Count

**FIGURE 6:**
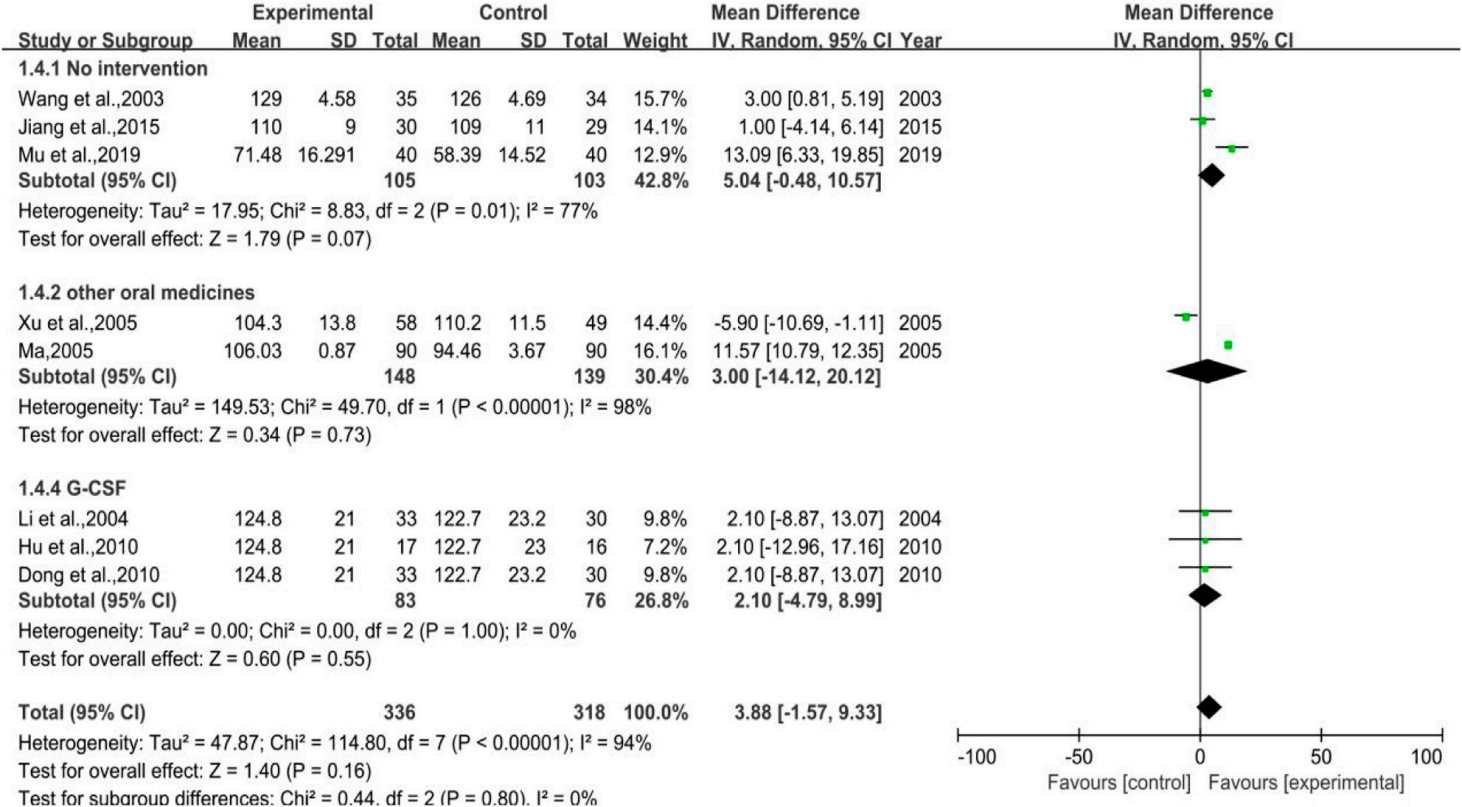
The forest plot of hemoglobin count in preventive medication.

The meta-analysis of hemoglobin count (shown in Figure 6) took altogether seven trials into account with 336 cases in treatment group and 318 cases in control group. Among all 7 trials, 3 were categorized in Subgroup 1, 2 belonged to Subgroup 2, and the rest 3 trials belonged to Subgroup 3. The heterogeneity results were *p* < 0.00001, I^2^ = 94% in total, *p* = 0.01, I^2^ = 77% in Subgroup 1, *p* < 0.00001, I^2^ = 98% in Subgroup 2, *p* = 1.00, and I^2^ = 0% in Subgroup 3, respectively. The meta-analysis result of total was [MD = 3.88, 95%CI (-1.57, 9.33)], Z = 1.40 (*p* = 0.16), and that of Subgroup 1 was [MD = 5.04, 95%CI (-0.48, 10.57)], Z = 1.79 (*p* = 0.07). The results of Subgroup 2 and Subgroup 3 were [MD = 3.00, 95%CI (−14.12, 20.12)], Z = 0.34 (*p* = 0.73) and [MD = 2.10, 95%CI (-4.79, 8.99)], Z = 0.60 (*p* = 0.55), respectively. DYT performed better in raising hemoglobin count, but not statistically significant.

#### 3.3.5 White Blood Cell Suppression Rate

##### 3.3.5.1 White Blood Cell Suppression Rate

**FIGURE 7:**
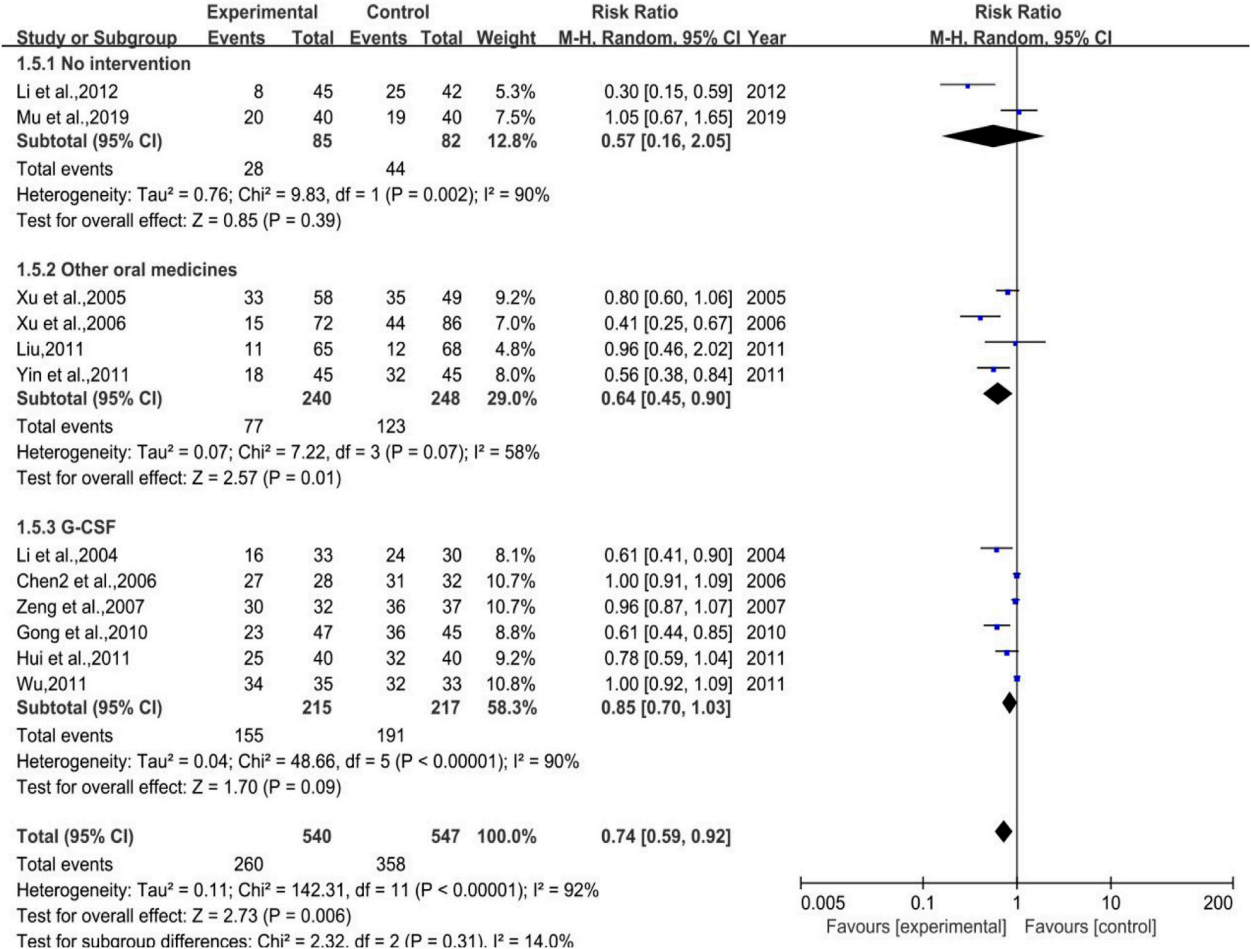
The forest plot of white blood cell suppression rate in preventive medication.

As shown in Figure 7, a total of 12 trials (2 in Subgroup 1, 4 in Subgroup 2, and 6 in Subgroup 3) were included in the meta-analysis of white blood cell suppression rate with the number of cases in treatment group and control group of 540 and 547, respectively. The heterogeneity results were as follows: *p* < 0.00001, I^2^ = 92% in total, *p* = 0.002, I^2^ = 90% in Subgroup 1, *p* = 0.07, I^2^ = 58% in Subgroup 2. Meta regression showed that the heterogeneity of group 2 was not significantly related to the publication years (*p* = 0.297), the duration of medication (*p* = 0.238), the number of case (*p* = 0.939), anti-tumor treatment (*p* = 0.760), the dose of DYT (*p* = 0.431), study region (*p* = 0.123), and hospital grade (*p* = 0.207); *p* < 0.00001, I^2^ = 90% in Subgroup 3. Meta regression showed that there was no significant correlation between the heterogeneity of group 3 and the years of publication (*p* = 0.543), the duration of medication (*p* = 0.102), the number of case (*p* = 0.134), study region (*p* = 0.982), and hospital grade (*p* = 0.442). While the meta-analysis results were [RR = 0.74, 95%CI (0.59, 0.92)], Z = 2.73 (*p* = 0.006) in total, [RR = 0.57, 95%CI (0.16, 2.05)], Z = 0.85 (*p* = 0.39) in Subgroup 1, [RR = 0.64, 95%CI (0.45, 0.90)], Z = 2.57 (*p* = 0.01) in Subgroup 2, sensitivity analysis showed that the results were stable, and the removal of each study had little effect on the overall results (Figure 17C), and Egger’s test (*p* = 0.739) showed that the included studies had no significant publication bias: [RR = 0.83, 95%CI (0.70, 1.03)], Z = 1.70 (*p* = 0.09) in Subgroup 3. Sensitivity analysis showed that the results were stable, and the removal of each study had little effect on the overall results (Figure 17D), and Egger’s test (*p* = 0.173) showed that there was no significant publication bias in the included studies. Overall, the efficacy of DYT was significantly superior to the control group in improving white blood cell suppression rate.

##### 3.3.5.2 III-IV Degree White Blood Cell Suppression Rate

**FIGURE 8:**
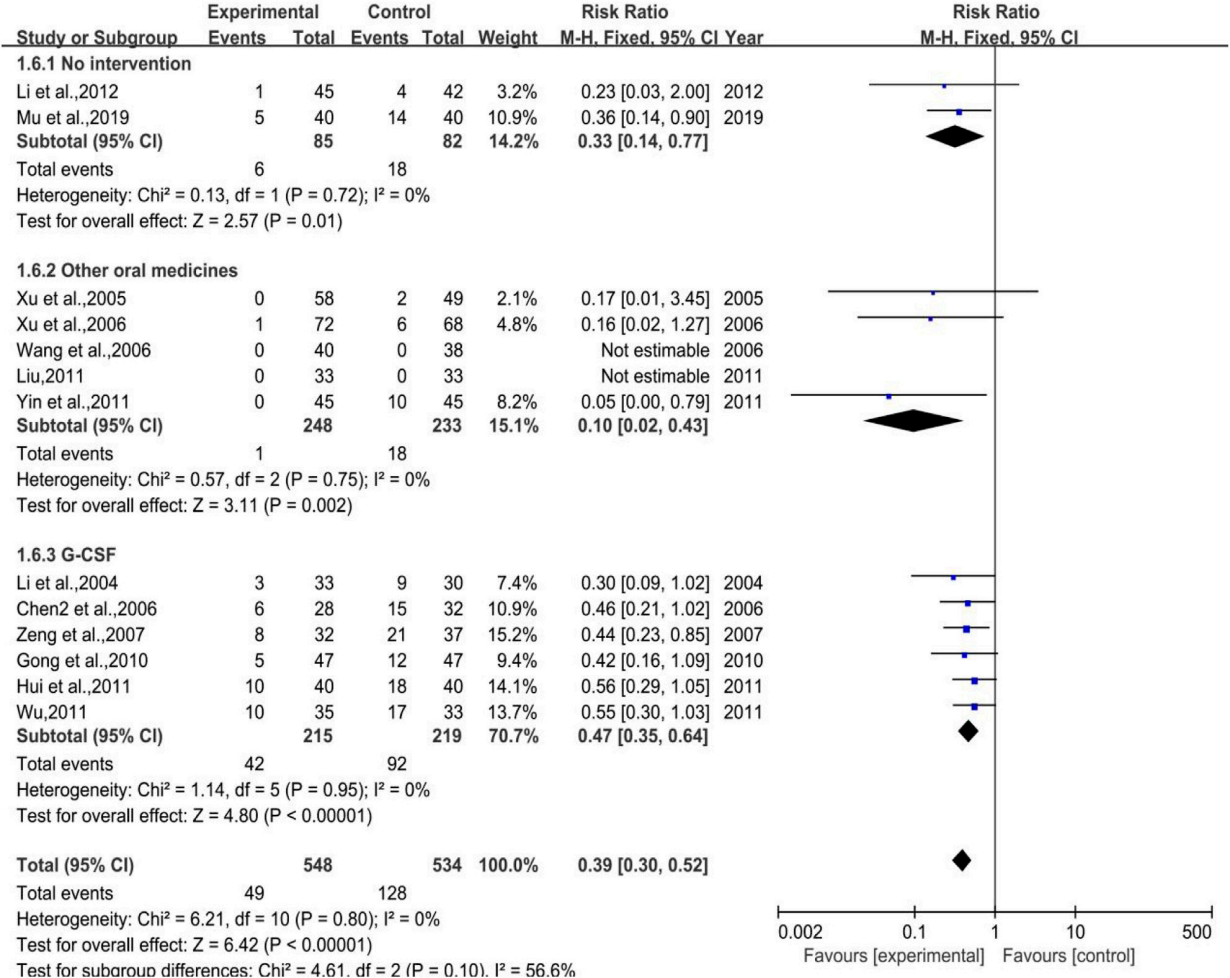
The forest plot of III-IV degree white blood cell suppression rate in preventive medication.

As shown in Figure 8, a total of 13 trials (2 in Subgroup 1, 5 in Subgroup 2, and 6 in Subgroup 3) were included in the meta-analysis of white blood cell suppression rate with the number of cases in treatment group and control group of 548 and 534, respectively. All the subgroups showed a low heterogeneity with *p* = 0.80, I^2^ = 0% in total, *p* = 0.72, I^2^ = 0% in Subgroup 1, *p* = 0.75, I^2^ = 0% in Subgroup 2, and *p* = 0.95, I^2^ = 0% in Subgroup 3, while the meta-analysis results were [RR = 0.39, 95%CI (0.30, 0.52)], Z = 6.42 (*p* < 0.00001) in total, [RR = 0.33, 95%CI (0.14, 0.77)], Z = 2.57 (*p* = 0.01) in Subgroup 1, [RR = 0.10, 95%CI (0.02, 0.43)], Z = 3.11 (*p* = 0.002) in Subgroup 2, and [RR = 0.47, 95%CI (0.35, 0.64)], Z = 4.80 (*p* < 0.00001) in Subgroup 3. Sensitivity analysis showed that the results were stable, and the removal of each study had little effect on the overall results (Figure 17E), and Egger’s test (*p* = 0.023) showed that the included studies may have some publication bias. Overall, the efficacy of DYT was significantly superior to the control group in improving III-IV degree white blood cell suppression rate.

#### 3.3.6 Application Amount of Granulocyte Colony-Stimulating Factor

**FIGURE 9:**
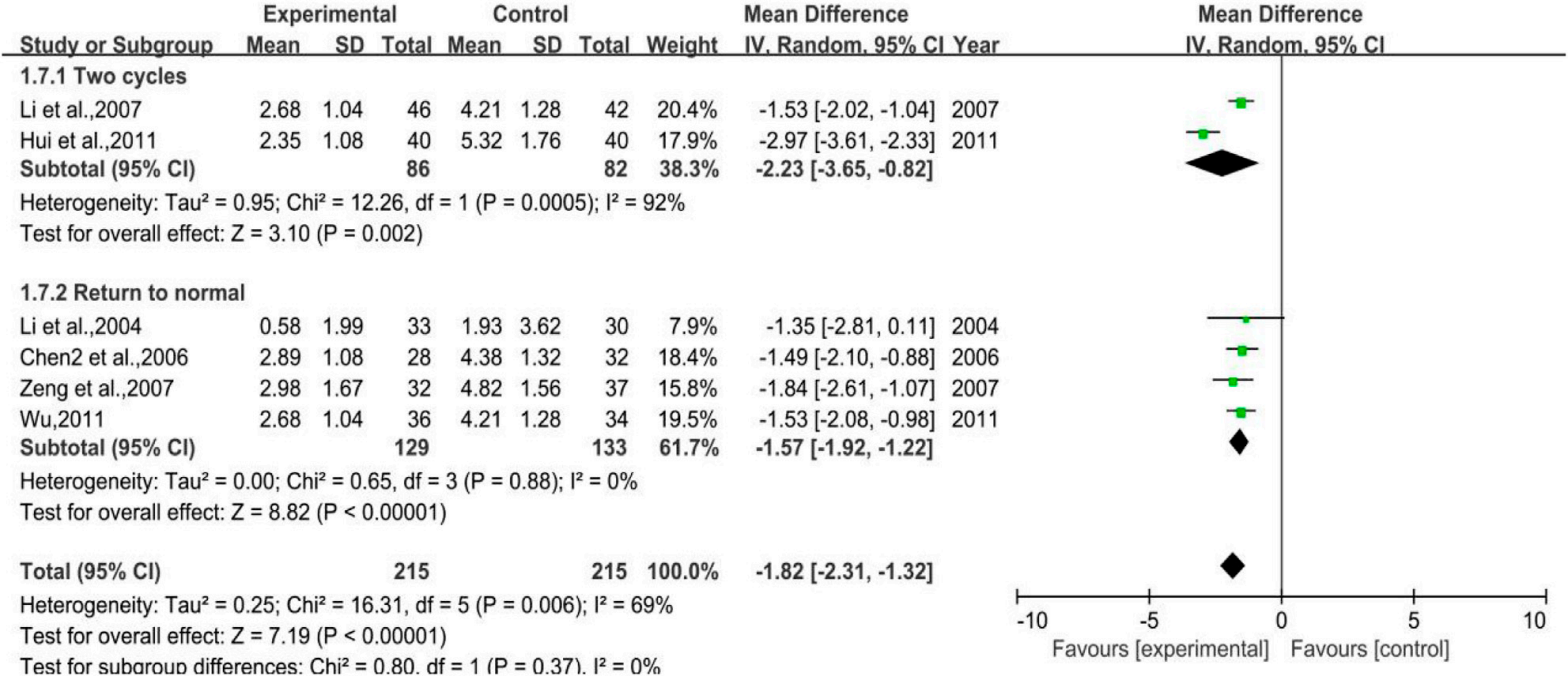
The forest plot of the application amount of G-CSF in preventive medication.

All the studies enrolled in the analysis were studied with a control group provided with G-CSF when needed and a treatment group using additional DYT based on the control group. In total, 4 trials were included for the outcome of WBC backing to the normal range, with 129 cases in the treatment group and 133 cases in the control group, while 2 trials were included for the outcome of 2 chemoradiotherapy periods with 86 and 82 cases in the treatment and control groups, respectively. The heterogeneity test and meta-analysis results are demonstrated in Figure 9. The result of the former outcome was *p* = 0.0005, I^2^ = 92% and [MD = −2.23, 95%CI (−3.65, −0.82)], Z = 3.10 (*p* = 0.002), while the latter one was *p* = 0.88, I^2^ = 0% and [MD = −1.57, 95% CI (−1.92, −1.21)], Z = 8.82 (*p* < 0.00001), sensitivity analysis showed that the results were stable, and each study had little effect on the overall results after removal (Figure 17F), and Egger’s test (*p* = 0.910) showed that the included studies had no significant publication bias. All the results above implied that further use of DYT can reduce the application amount of G-CSF by 1.57 and 2.23, respectively, according to two different treatment periods.

#### 3.3.7 CD3^+^ and CD4^+^

**FIGURE 10:**
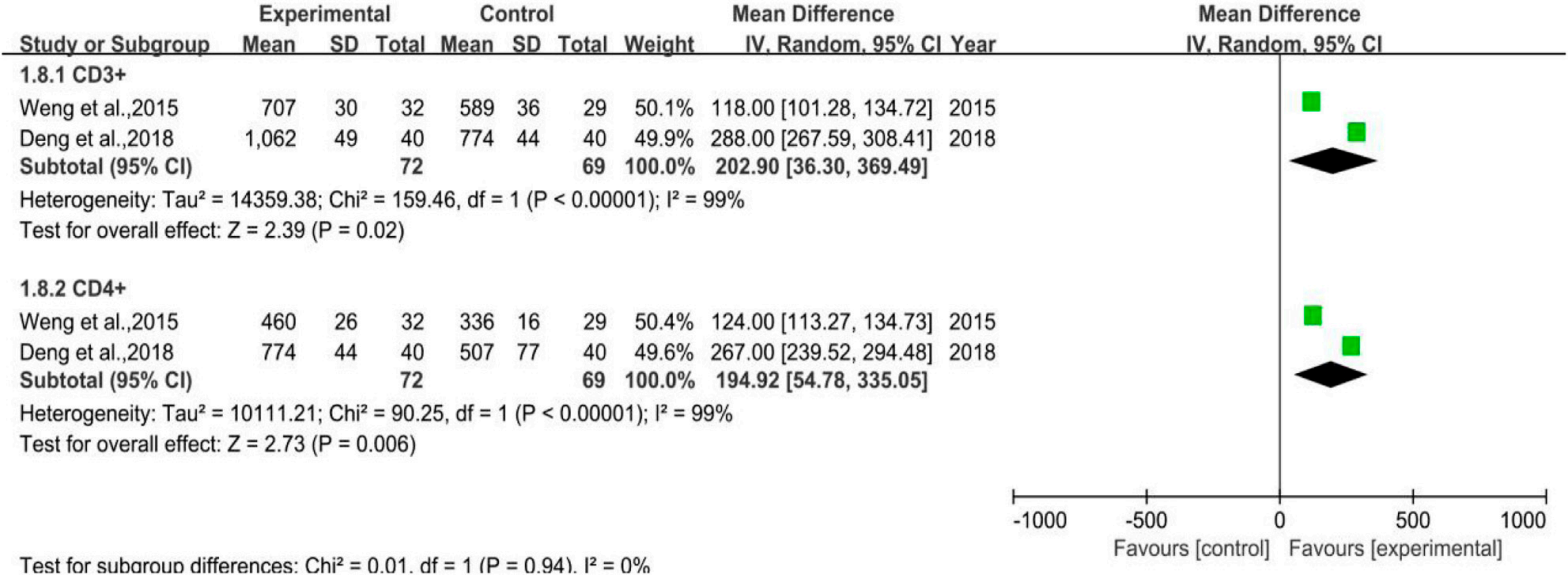
The forest plot of CD3^+^ and CD4^+^ in preventive medication.

A total of 2 trials were included without any WBC-elevating drugs (Subgroup 1), the number of cases in the treatment group and control group were 72 and 69, respectively. The heterogeneity test and meta-analysis results of CD3^+^ were *p* = 0.0005, I^2^ = 92% and [MD = 202.90, 95%CI (36.30, 369.49)], Z = 2.39 (*p* = 0.02), while that of CD4^+^ were *p* < 0.00001, I^2^ = 99% and [MD = 194.92, 95%CI (54.78, 335.05)], Z = 2.73 (*p* = 0.006), which inferred that DYT may help improve body immunity for preventive medication purpose. All the results and the forest plot are displayed in Figure 10.

#### 3.3.8 ORR and DCR

**FIGURE 11:**
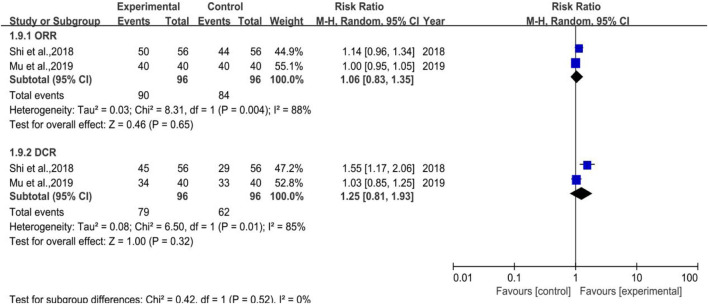
The forest plot of ORR and DCR in preventive medication.

There were 2 trials enrolled (depicted in Figure 11), which belonged to Subgroup 1, with 96 cases in both the treatment and control groups. The heterogeneity test of DCR was *p* = 0.01, I^2^ = 85% and its meta-analysis result showed that [RR = 1.25, 95%CI (0.81, 1.93)], Z = 1.00 (*p* = 0.32), which indicated that DYT can ameliorate the efficacy in solid tumor while without statistical significance. As a result, more pertinent researches remain to be included to verify the results.

### 3.4 Meta-Analysis Results of Therapeutic Medication

#### 3.4.1 White Blood Cell Count

**FIGURE 12:**
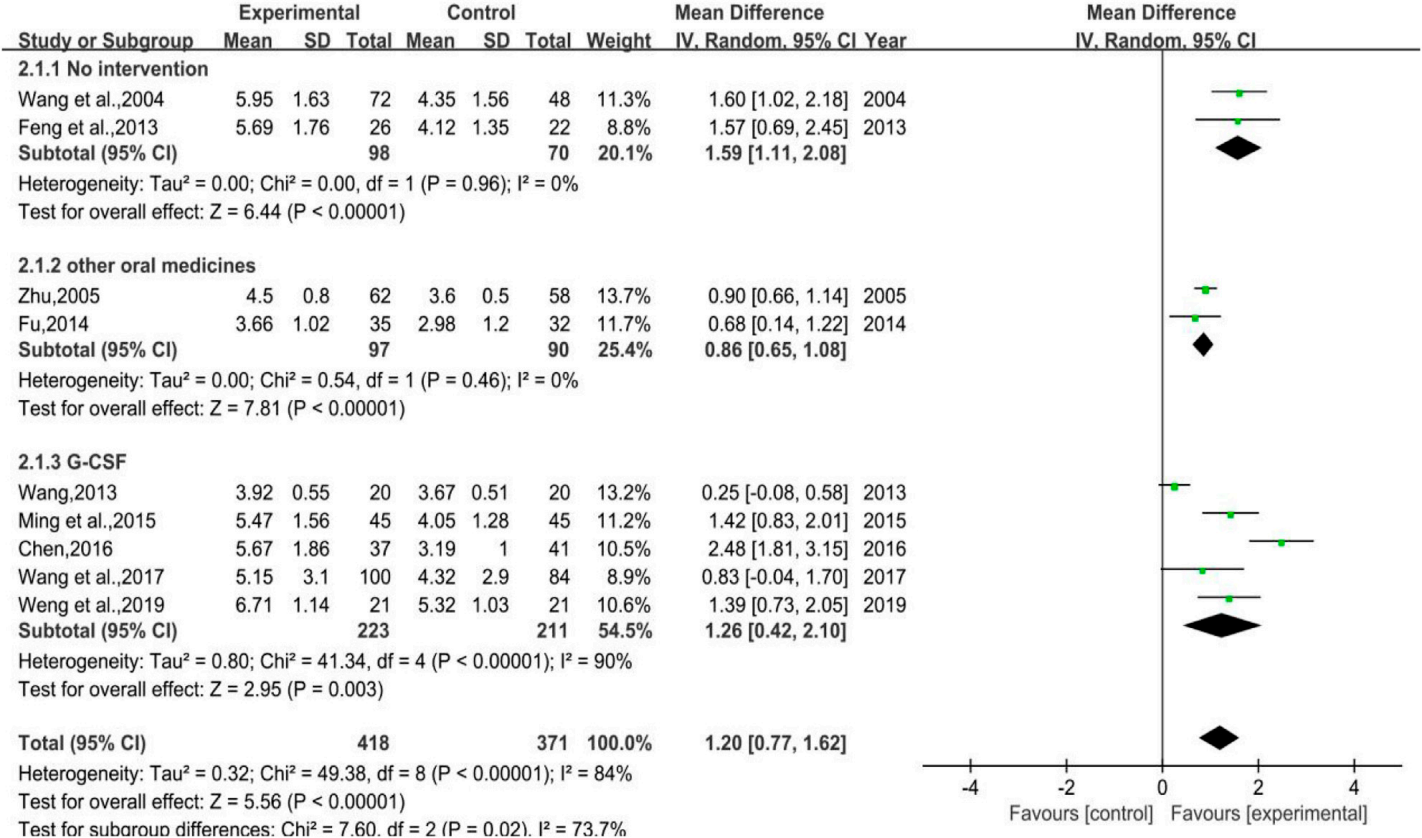
The forest plot of white blood cell count in therapeutic medication.

A total of 9 trials were included, of which 2 were with no-treatment control group (Subgroup 1), another 2 were control group with other WBC-elevating drugs (Subgroup 2), and the rest were with G-CSF (Subgroup 3). The number of cases in treatment group and control group were 418 and 371, respectively. The heterogeneity varied in different groups for *p* < 0.00001, I^2^ = 84% in total; *p* = 0.96, I^2^ = 0% in Group 1; *p* = 0.46, I^2^ = 0% in Group 2; *p* < 0.00001, I^2^ = 90% in Group 3, meta regression showed that the heterogeneity of Group 3 was not significantly correlated with the publication years (*p* = 0.576), the duration of medication (*p* = 0.352), the number of cases (*p* = 0.397), anti-tumor treatment (*p* = 0.432), the dose of DYT (*p* = 0.426), and study region (*p* = 0.358). The results of meta-analysis showed that [MD = 1.20, 95%CI (0.77, 1.62)], Z = 5.56 (*p* < 0.00001) in total; [MD = 1.59, 95%CI (1.11, 2.08)], Z = 6.44 (*p* < 0.00001) in Group 1; [MD = 0.86, 95%CI (0.65, 1.08)], Z = 7.81 (*p* < 0.00001) in Group 2; [MD = 1.26, 95%CI (0.42, 2.10)], Z = 2.95 (*p* = 0.003) in Group 3. Sensitivity analysis showed that the results were stable, and the removal of each study had little effect on the overall results (Figure 17G). Also, Egger’s test (*p* = 0.141) showed that the included studies had no significant publication bias. The results implied that for therapeutic medication, the efficacy of DYT in improving WBC count was superior to the control group. The forest plot is shown in Figure 12.

#### 3.4.2 Neutrophile Count

**FIGURE 13:**
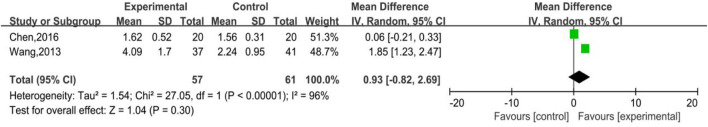
The forest plot of neutrophile count in therapeutic medication.

As shown in Figure 13, there were 2 trials involved in the analysis, which were parts of Subgroup 3, with 57 and 61 number of cases in the treatment group and control group, respectively. The heterogeneity (*p* < 0.00001, I^2^ = 96%) and meta-analysis results [MD = 0.93, 95%CI (-0.82, 2.69), Z = 1.04 (*p* = 0.30)] revealed that for therapeutic medication, DYT was better than other therapies in raising neutrophile count while without statistical significance.

#### 3.4.3 White Blood Cell Suppression Rate

##### 3.4.3.1 White Blood Cell Suppression Rate

**FIGURE 14:**
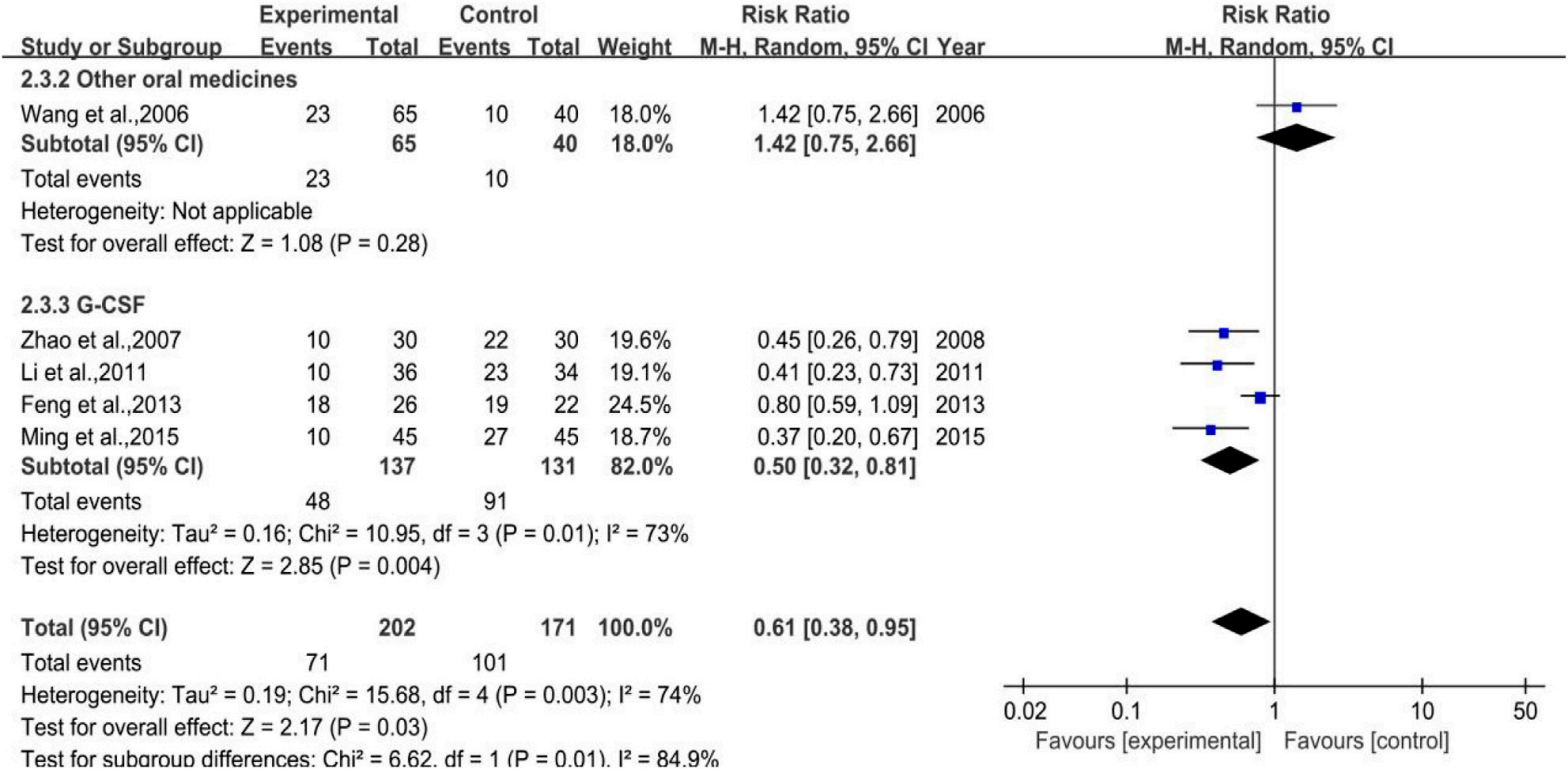
The forest plot of white blood cell suppression rate in therapeutic medication.

A total of 5 trials (1 in Subgroup 1, 4 in Subgroup 3) were included in the meta-analysis of white blood cell suppression rate with 202 and 171 number of cases in the treatment group and control group, respectively. The heterogeneity results were *p* = 0.003, I^2^ = 74% in total, while that of Subgroup 3 were *p* = 0.01, I^2^ = 73%, meta-regression showed that the heterogeneity of group 3 was not significantly in correlation with the years of publication (*p* = 0.880), the duration of medication (*p* = 0.888), the number of cases (*p* = 0.146), anti-tumor treatment (*p* = 0.359), the dose of DYT (*p* = 0.109), and study region (*p* = 0.945). Besides, the meta-analysis results (shown in Figure 14) were [RR = 0.61, 95%CI (0.38, 0.95)], Z = 2.17 (*p* = 0.03) in total and [RR = 0.50, 95%CI (0.32, 0.82)], Z = 2.85 (*p* = 0.004) in Subgroup 3. Sensitivity analysis showed that the results were stable, and the removal of each study had little effect on the overall results (Figure 17H), and Egger’s test (*p* = 0.003) showed that the included studies may have some publication bias. Overall, for therapeutic medication purpose, the efficacy of DYT was significantly superior to the control group in improving the white blood cell suppression rate.

##### 3.4.3.2 III-IV Degree White Blood Cell Suppression Rate

**FIGURE 15:**
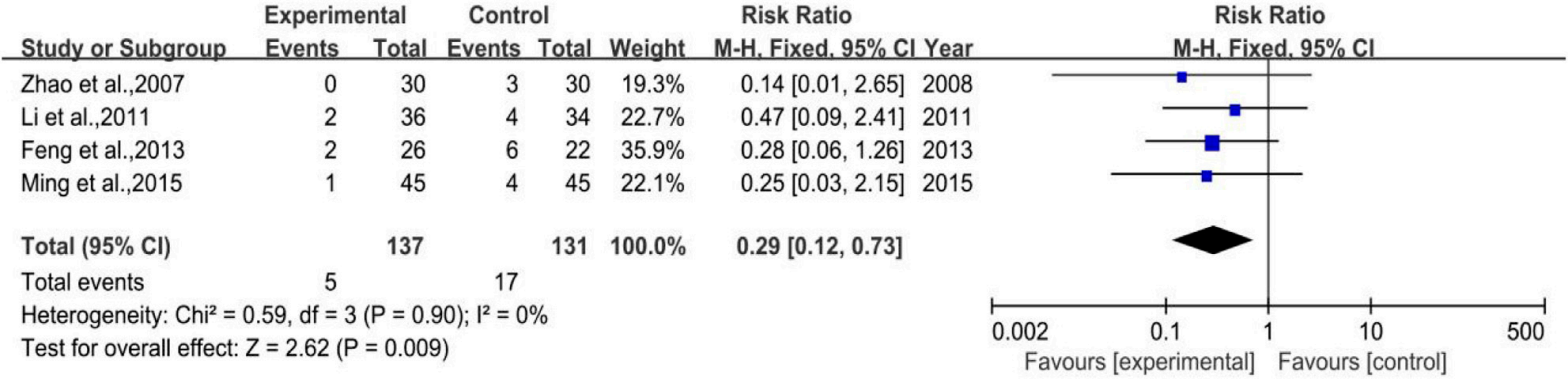
The forest plot of III-IV degree white blood cell suppression rate in therapeutic medication.

All 4 trials enrolled in the analysis were studied with the control group provided with G-CSF (Subgroup 3) with 137 and 131 number of cases in the treatment group and control group, respectively. The heterogeneity and meta-analysis results are shown in Figure 15, which were *p* = 0.90, I^2^ = 0% and [RR = 0.29, 95%CI (0.12, 0.73)], Z = 2.62 (*p* = 0.009). Sensitivity analysis showed that the results were stable, and the removal of each study had little effect on the overall results (Figure 17I), and Egger’s test (*p* = 0.285) showed that the included studies had no significant publication bias. In general, for therapeutic medication purpose, the efficacy of DYT was significantly superior to the control group in improving III-IV degree white blood cell suppression rate.

#### 3.4.4 White Blood Cell Effective Rate

**FIGURE 16:**
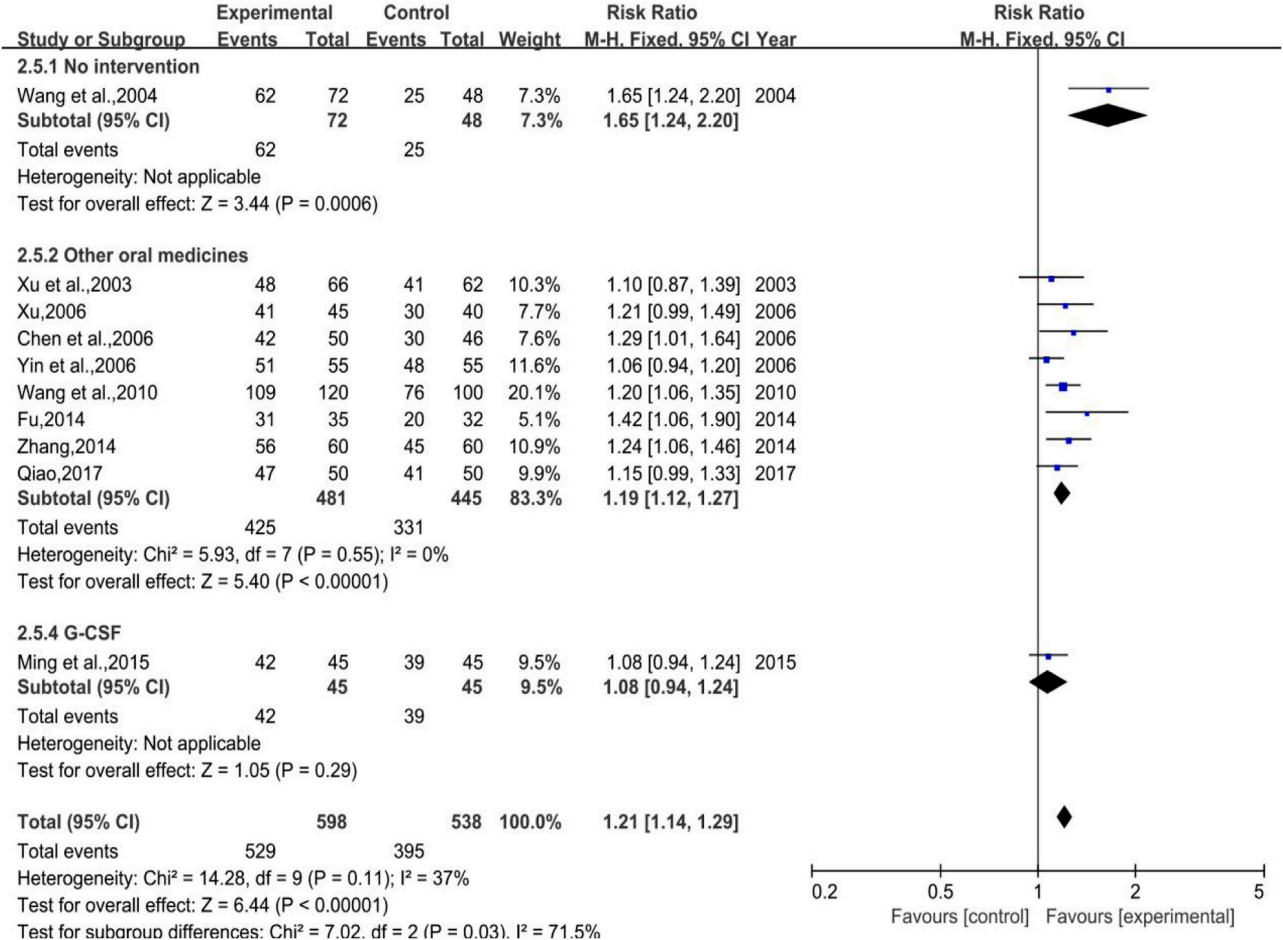
The forest plot of white blood cell effective rate in therapeutic medication.

**FIGURE 17:**
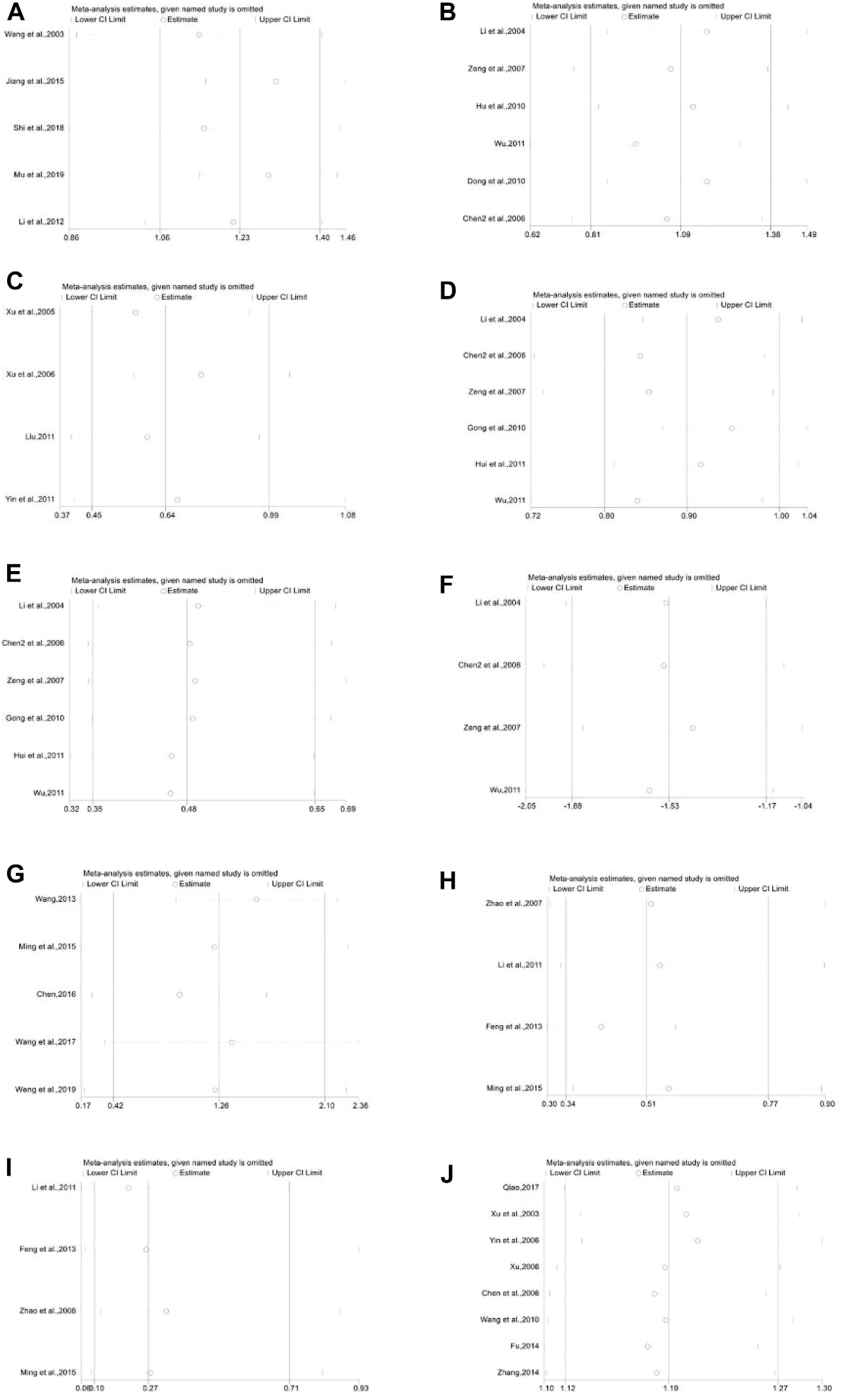
Sensitivity analysis plot. **(A)**: White blood cell count of subgroup 1 in preventive medication, **(B)**: White blood cell count of subgroup 3 in preventive medication, **(C)**: White blood cell suppression rate of subgroup 2 in preventive medication, **(D)**: White blood cell suppression rate of subgroup 3 in preventive medication, **(E)**: III-IV degree white blood cell suppression rate of subgroup 3 in preventive medication, **(F)**: Application amount of G-CSF, **(G)**: White blood cell count of subgroup 3 in therapeutic medication, **(H)**: White blood cell suppression rate of subgroup 3 in therapeutic medication, **(I)**: III-IV degree white blood cell suppression rate of subgroup 3 in therapeutic medication, **(J)**: White blood cell effective rate of subgroup 2 in therapeutic medication.

A total of 10 trials (1 in Subgroup 1, 8 in Subgroup 2, and the rest in Subgroup 3) were included with 598 and 538 number of cases in the treatment group and control group, respectively.. A low heterogeneity showed both in total (*p* = 0.11, I^2^ = 37%) and in Subgroup 2 (*p* = 0.55, I^2^ = 0%), while the meta-analysis results were [RR = 1.21, 95%CI (1.14, 1.29)], Z = 6.44 (*p* < 0.00001) in total and [RR = 1.19, 95%CI (1.12, 1.27)], Z = 5.40 (*p* < 0.00001) in Subgroup 2 (shown in Figure 16). Sensitivity analysis showed that the results were stable, and the removal of each study had little effect on the overall results (Figure 17J), and Egger’s test (*p* = 0.126) showed that the included studies had no significant publication bias, which inferred that DYT was better than the control group in raising white blood cell effective rate with statistically significant difference between the two groups.

### 3.5 Occurrence of Adverse Events

Among all the 41 trials, there were 26 trials which failed to report the occurrence of adverse events, while another 11 reported no obvious adverse events. Hong Xu et al.’s research (Xu, 2006) reported 2 cases of mild stomach discomfort, and the same adverse events appeared in 3 patients in the study of Zhaoyu Zeng et al. (2007); The study of Jianyi Gong et al. (Gong and Duan, 2010) reported 13 cases of upper abdominal discomfort and 5 of acid reflux; In Daying Mou et al.’s study (Mou et al., 2019), there were 22 and 20 cases of different gastrointestinal reactions, respectively, 9 and 10 cases of hepatic impairment as well as 3 and 4 cases of renal impairment.

## 4 Discussion

Sanguisorbae Radix has a long history of medicinal use, which was first recorded in “Shen Nong’s Herbal Classic” (Wu, 2011): “Bitter taste and slightly cold in nature, it has a marked effect in treating women with spasms and cramps during childbirth, various debilitating diseases, and collateral diseases. It has the effects of relieving pain, removing carrion, antiperspirant, and curing metal wounds,” Though classified as blood-cooling and blood-stanching in modern Chinese medicine, it also has records of generating and nourishing blood in the ancient Chinese herbal classics, such as “New Compilation of Materia Medica” (Qing and Chen SD, 1996): “Some people feel confused that given its blood-cooling nature, how can Sanguisorbae Radix stop bleeding? They don’t know it can also nourish blood...” “A Readable Classic of Materia Medica” (Qiang and Wang RA, 1987)“Sanguisorbae Radix tastes bitter and sour and is slightly cold in nature...It can cure all kinds of blood loss...,” “Annotation to Shen Nong’s Herbal Classic” (Qiang and Zhang ZC, 1992): “Sanguisorbae Radix, also known as jade bean... can nourish the blood of liver.”

Modern pharmacologic research found that active ingredients of Sanguisorbae Radix include saponin, flavone, tannin, etc., of which, Sanguisorbae Radix saponin is proven to promote the hematopoiesis of bone marrow. One mechanism is that it promotes the proliferation of bone marrow stromal cells and improves and stabilizes the hematopoietic microenvironment. The other attributes to its function of promoting hematopoietic cells’ proliferation and differentiation by facilitating the production of hematopoietic growth factors (HGFs) as well as simultaneously enhancing the expression of HGFs’ receptors (C-KIT, IL-3 receptors, TPO receptors, etc.) (Gao et al., 2006; Zou et al., 2012; Dai et al., 2014; Dai et al., 2016).

### 4.1 Main Findings

A total of 41 studies were enrolled in our research, involving 3,793 cases, ensuring sample size sufficiency. For raising white blood cell count, the efficacy of DYT for both preventive and therapeutic purposes was significantly superior to any other control group. The superiority over the control group with no treatment overshadowed the control group with other WBC-elevating drugs or G-CSF when needed. Sensitivity analysis and Egger’s test showed that the results were objective and stable. For the improvement of neutrophil count, it was found that the Diyu Shengbai tablet was more effective than other WBC-elevating drugs or G-CSF when needed. However, with limited studies and cases being included, more high-quality studies are needed to verify the efficacy of neutrophil count. To improve the white blood suppression rate, additional use of DYT helps reduce the total suppression rate and III-IV degree suppression rate of WBC. The difference was statistically significant. As for blood platelet, DYT can increase the blood platelet count while decreasing the suppression rate with a superiority to the control group with no treatment and other WBC-elevating drugs. Similarly, its superiority to G-CSF when needed remains to be verified for limited studies and cases being included.

For the improvement of platelet and hemoglobin count, DYT had a certain effect on them. However, except for the control group without treatment, the superiorities to other therapies were not significant. In addition, using DYT can reduce the application amount of G-CSF, and the differences are statistically significant. Only 4 studies reported the occurrence of adverse events, which mainly concentrated on mild gastrointestinal and hepatorenal events that mostly can be diminished or relieved after clinical treatment. Due to patients being treated with radiotherapy or chemotherapy, the reason for the occurrence of adverse events cannot be identified.

### 4.2 Comparisons to the Previous Meta-Analysis

There are two previous studies about meta-analysis which have estimated the clinical efficacy of DYT being used for treating leukopenia induced by radiotherapy and chemotherapy. The research of Rui Zhang et al. took 8 studies into account and the clinical outcomes only involved the WBC suppression rate (Zhang et al., 2012), while the other research of Zefeng Zhao et al. included 12 studies in total with clinical outcomes of myelosuppression rate, WBC count, and the amount of G-CSF. It may have a negative impact on the reliability of the results for their limited number of literatures. and small sample size enrolled, as well as the low quality of included researches (randomized and quasi-randomized trials were included) and no subgroup analysis according to different treatment methods.

In contrast, a comprehensive retrieval was conducted, and strict criteria of inclusion and exclusion were set in our study. More importantly, we enrolled some recently published research and performed subgroup analysis in order to improve the methodology and strengthen the stability of the results. Also, we performed subgroup analysis according to different treatments of rising white blood cells and performed meta-regression and sensitivity analysis for the clinical outcomes with more considerable heterogeneity to find the source of heterogeneity, while we did not find it. But we discovered that outcomes with significant heterogeneity were obtained for the reason that there were few studies included (CD3^+^, CD4^+^, ORR, DCR, etc.). It might reduce heterogeneity test efficiency in meta-analysis. Besides, some clinical outcomes (platelet counts, hemoglobin counts, etc.) could usually be exaggerated statistical variations when evaluated as measurement data due to a larger range of average clinical values. So we speculated that the results of studies which showed the heterogeneity in statistics might be related to the clinical heterogeneity. It meant that different illness degrees (specific white blood cell counts) and other different clinical features (tumor types, pathological stages, etc.) might be one of the sources of heterogeneity in meta-analysis.

### 4.3 Limitations

Several limitations still exist in our study. First, only 8 of 41 literatures reported specific randomized methods. As the years of publication for some original studies were too early, and many studies did not report the use of allocation concealment and blinding, their quality was not high. However, this study aimed to discuss the effect of the Diyu Shengbai tablet on leukopenia. We chose objective outcomes of clinical laboratories, such as WBC counts and NEUT counts, with little influence from allocation concealment and blinding. Second, although the white blood cell counts of patients at the time of enrollment have been divided into preventive medication and therapeutic medication, there are still some differences in the white blood cell counts among the included studies. Third, the heterogeneity among studies could not be neglected owing to different interventions, drug doses, and therapy time applied to patients with different WBC levels in each trial. In our research, we conducted a subgroup analysis to diminish the heterogeneity to some extent. Finally, as for adverse events, due to the limited follow-up time of included studies and unstandardized reports of some research, no definite conclusions can be drawn about the adverse reactions of DYT.

## 5 Conclusion

DYT do have positive effect on preventing and treating leukopenia caused by radiotherapy and chemotherapy against malignant tumor. Its efficacy is superior to Leucogen tablets and Batilol tablets, and the application amount of G-CSF can also be diminished while using it. For some clinical outcomes, larger sample size and well-designed randomized controlled trials were still needed to validate our conclusions further. Some of the literatures we screened were published too early, and the average quality and numbers of included literatures were limited.

## Data Availability

The original contributions presented in the study are included in the article/Supplementary Material. Further inquiries can be directed to the corresponding authors.
